# Understanding Stabilization of Oil-in-Water Emulsions with Pea Protein—Studies of Structure and Properties

**DOI:** 10.1021/acs.langmuir.4c00540

**Published:** 2024-06-21

**Authors:** Eleonora Olsmats, Adrian R. Rennie

**Affiliations:** Macromolecular Chemistry, Department of Chemistry—Ångström, Uppsala University, Box 538, 75121 Uppsala, Sweden

## Abstract

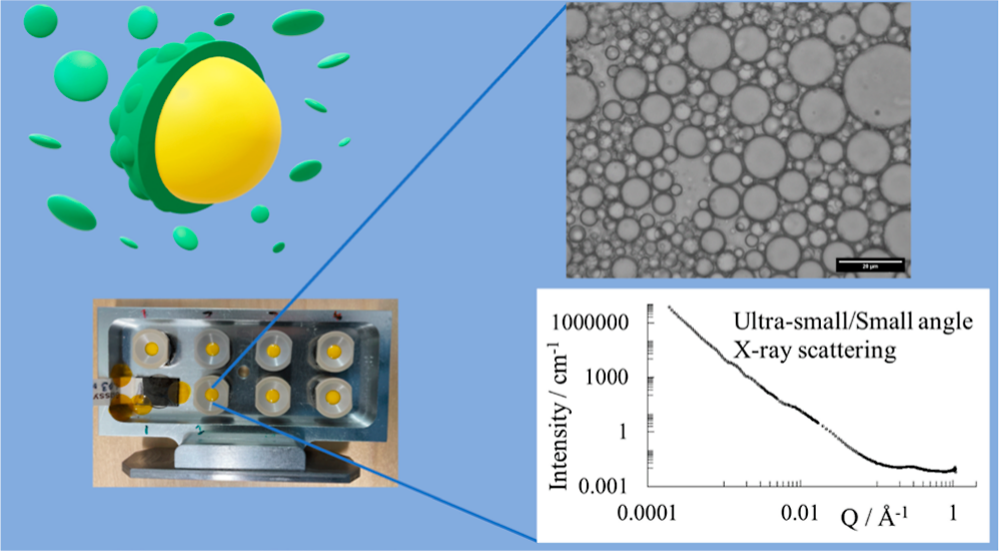

This study investigates the stability and structure of oil-in-water emulsions stabilized by pea protein. Of the wide range of emulsion compositions explored, a region of stability at a minimum of 5% w/v pea protein and 30–50% v/v oil was determined. This pea protein concentration is more than what is needed to form a layer covering the interface. X-ray scattering revealed a thick, dense protein layer at the interface as well as hydrated protein dispersed in the continuous phase. Shear-thinning behavior was observed, and the high viscosity in combination with the thick protein layer at the interface creates a good stability against creaming and coalescence. Emulsions in a pH range from acidic to neutral were studied, and the overall stability was observed to be broadly similar independently of pH. Size measurements revealed polydisperse protein particles. The emulsion droplets are also very polydisperse. Apart from understanding pea protein-stabilized emulsions in particular, insights are gained about protein stabilization in general. Knowledge of the location and the role of the different components in the pea protein material suggests that properties such as viscosity and stability can be tailored for various applications, including food and nutraceutical products.

## 1 Introduction

Emulsions have many applications such as in the food, pharmaceutical, and chemical industries.^1,2^ Considering emulsion systems for food applications, pea protein has been identified as an interesting candidate as a stabilizer.^3^ A summary of the reported pea protein emulsions with various stabilization mechanisms is provided in our recent review.^4^ The largest fraction of pea protein is globulins (65–80%) followed by albumins (10–20%).^5^ The amino acid composition has been reported, e.g., by Pownall et al.^6^ and by Ma et al.,^7^ but compositional differences are common depending on external factors such as local variety, season variability, and growing conditions. While there have been several attempts to make particle-stabilized emulsions with protein-based materials such as pea protein,^8^ soy protein,^9^ and whey protein,^10^ primarily low-molecular-weight emulsifiers such as surfactants or lipids, or amphiphilic biopolymers and especially proteins are used.^11^ The protein stabilizers form viscoelastic layers on the emulsion droplets and also modify the interfacial tension. Particle-stabilized emulsions, or Pickering emulsions, attract increased attention, as they provide a barrier to destabilization with slow rates of removal from the interface. The detachment energy, per unit area of surface, to remove a solid colloidal particle that is absorbed at the interface is very high and is given by
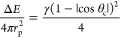
1where *r*_p_ is the particle radius, γ is the interfacial tension between the two phases, and θ_c_ is the contact angle of the particle.^12^ The contact angle is strongly dependent on the material hydrophobicity, and low solubilities in both the aqueous and oil phases are favorable characteristics for Pickering particles. This detachment energy can be much larger than that of typical thermal energies. Particle stabilization of emulsions with aggregates of pea protein has been suggested, for example, in the studies of Li et al.^13^ and Velandia et al.,^14^ but there are indications of differences with pH. Other studies, such as those of Sridharan et al.,^15^ have suggested the stability to be mostly related to the contribution of individual protein molecules. Their conclusions are based on comparison of experimental data and calculated surface coverage, where in the experiments, individual protein molecules could cover 47% of the interface but larger particles would cover just 3% in their formulations. Pea protein possesses properties that are comparable to both particle-like Pickering stabilizers and amphiphilic polymers. An important aspect of these two different ways of contributing to stabilization is the formation of an interfacial layer, which can give rise both to steric effects and to electrostatic interactions. The interfacial properties, such as protein migration and adsorption at the interface, are important. Additionally, the role of non-surface-active pea protein material will be discussed in this article.

Applications of emulsions often need a range of physical properties: stability under storage for up to 1 year may be required for processed food products. Rheological behavior is important for many uses. It is also useful to establish over what range of compositions emulsions can be prepared. Although there is extensive literature on emulsions with pea proteins that has been reviewed recently,^4^ it is valuable to explore a broader range of compositions and pH and to determine information about the structural arrangement of dispersed pea protein in the emulsions.

In outline, after a description of the experimental methods and materials, the paper reports the observations of stability as a function of oil fraction, protein amount, and pH by direct optical observation of phase behavior with time. This is followed by characterization of the protein isolate in the aqueous phase without oil. Small angle X-ray scattering (SAXS) results are then presented and used to provide an overall structural model of the emulsion. Some rheological properties from low amplitude oscillatory shear and continuous shear measurements are then reported that can be correlated to these results. Finally, conclusions and an outlook are presented.

## 2 Experimental Section

### 2.1 Materials

Pea [*Pisum sativum* L.] protein was purchased from Superfruit Scandinavia AB (Växjö, Sweden). According to the manufacturer, the protein content was 83% w/w. Rapeseed oil was supplied by Di Luca & Di Luca AB (Stockholm, Sweden). All other chemicals were of analytical grade. Citric acid and sodium citrate were obtained from Sigma-Aldrich (St. Louis, MO, USA).

### 2.2 Characterization of Protein Dispersions in Water and Buffer Solutions

Pea protein dispersions of 0.01% w/v pea protein were prepared in citrate buffers with 0.1 M solutions according to the method of Gomori at pH 3.0–6.2.^16^ Citric acid and sodium citrate solutions were mixed in appropriate ratios to obtain the desired pH and pea protein was dispersed in the buffer solutions. A 0.45 μm filter was used before the measurements. The surface potential of the pea protein was determined using a Zetasizer Pro (Malvern Panalytical Ltd., Malvern, UK) with DTS1070 cells to measure the electrophoretic mobility of the prepared dispersions. A He–Ne (633 nm) laser source was used with a scattering angle of 173°, and the viscosities of the dilute dispersions were assumed to be that of water to calculate hydrodynamic radii. The zeta potential, ζ, was calculated using the Malvern software as
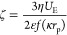
2where η is the viscosity of the sample, *U*_E_ is the electrophoretic mobility, ε is the dielectric constant, and *f*(κ*r*_p_) is Henry’s function that depends on the product of the inverse screening length κ and the particle radius *r*_p_ and is calculated in the Smoluchowski approximation as 1.5 for high ionic strength solutions. The experiments were performed after allowing for 60 s equilibration at a temperature of 25 °C and with three replicate measurements separated by 60 s. The data are reported by plotting the zeta potential versus sample pH, with error bars corresponding to differences in triplicate measurements. The instrument was further used to measure dynamic light scattering (DLS) and hence assess particle size. The refractive indices of the medium and the dispersed material were set to 1.33 and 1.45, respectively. The results are presented as the intensity and volume distributions of the particle radius, and the measurements are repeated ten times.

### 2.3 Physical Storage Stability of Emulsions

Series of emulsions were prepared with 0.5–20% w/v pea protein with various rapeseed oil fractions of 5–80% v/v. Emulsions were prepared by two different high-sheer methods: by using a Blender 2 Go, model BL3326B (Clas Ohlson AB, Insjön, Sweden) or by using a rotor stator D1000 homogenizer with D1000-M5, 5 × 50 mm flat bottom probe (Benchmark Scientific Inc., Edison, NJ, USA). Protein and rapeseed oil were mixed with deionized water or citrate buffer in appropriate ratios for 1 min. The sample volume prepared in the food blender was 100 mL in a 600 mL beaker and mixed at speed II. The sample volume prepared with the homogenizer was 1 mL in 2 mL microtubes and homogenized at speed 3 (∼17,100 rpm). The fresh homogenized emulsions were left standing and stored at 22 °C in 2 mL microtubes. The phase separation development was recorded continuously during storage, and the final visual observations after 7 days were recorded by photographs and plotted as ternary-phase diagrams of the stability. The emulsion stability was categorized by the formation of separated layers or visually homogeneous samples. The compositions were expressed as data points of mass percentage of oil, water, and 25% w/v pea protein solution in water. For example, a sample with 40% v/v oil, 60% v/v water, and 7.5% w/v pea protein would be represented in the center of the map, with composition 35.3% w/w oil, 35.9% w/w water, and 28.9% w/w pea protein solution in water.

### 2.4 Small/Ultra Small Angle X-ray Scattering Analysis of Emulsions

SAXS measurements of the emulsions were made using a Xeuss 2.0 QZoom (Xenocs, Grenoble, France) instrument at Uppsala University, Sweden. The Genix3D Cu ULD source produced the X-ray beam with wavelength 1.54 Å, collimated with apertures set at 0.7 mm × 0.7 mm near the source and 0.4 mm × 0.4 mm before the sample, and the scattered radiation was detected with a Pilatus 3R 300k detector (Dectris, Switzerland). For the SAXS measurements, the instrument was used in two configurations with sample to detector distances of 300 and 2400 mm. The samples were loaded into gel holders with circular apertures of approximately 2.5 mm diameter and sealed with Kapton windows to give approximately 1.5 mm sample thickness. The temperature was kept constant at 22 °C throughout the experiment, and the samples were held in vacuum during the measurements. The measurement times were 60 min at each detector distance. The SAXS data were reduced with the XSACT software (Xenocs).

Ultra small angle X-ray scattering (USAXS) measurements of the prepared emulsions were performed with the same samples in the gel holder as those for the SAXS measurements. The Bonse-Hart^17,18^ configuration was used and the beam was collimated with 3.0 mm × 3.0 mm and 1.4 mm × 1.4 mm apertures. Briefly, the X-ray beam was collimated with a Bartels channel-cut Si(111) crystal monochromator before hitting the sample and the angle of the scattered radiation was defined by a 4 bounce channel-cut Si(111) crystal analyzer in scanning operation mode. The measuring time for each sample was approximately 4 h. The USAXS scattering data were reduced in USAXSgui (Xenocs). For the merged curves, the desmeared USAXS data were scaled to the SAXS data, and the data were merged to the same value over the common data range.

The data are presented as logarithmic plots of intensity, *I*, versus the scattering vector, *Q*. The scattering vector is defined as
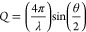
3where λ the wavelength and θ is the scattering angle. In scattering experiments, the *Q* vector represents the momentum transfer from the incoming wave to the scattered wave. The combined *Q* range for the SAXS and USAXS measurements was 0.0002–1 Å^–1^, based on calibration with silver behenate. The model fits^19^ were made separately on the slit smeared USAXS data and the pinhole smeared SAXS data to allow for the very different resolution and the fitting parameters were kept linked to identical values for the two data sets. The mass densities and scattering length densities of the components used for fitting purposes are listed in Table 1. Making fits to the SAXS data with an absolute scale of intensity was an important constraint on the parameters in the model. The intensity calibration was made by normalizing the scattering to that of the transmitted direct beam and checked by comparison to the measured scattering of glassy carbon.^20^

**Table 1:** Mass Density and Scattering Length Density for the Emulsion Components

component	mass density / g cm^–3^	scattering length density / 10^–6^ Å^–2^
H_2_O	0.997	9.41
rapeseed oil	0.916	8.68
pea protein	1.2^a^	11–12^b^

a The mass density of the pea protein was estimated from density measurements of the protein in solution.

b The scattering length density of the pea protein is highly dependent on the degree of hydration. The reported value is estimated from scattering model fits and comparable to similar proteins.

### 2.5 Rheology of Emulsions

The rheological behavior of the prepared emulsions was studied using a Modular Compact Rheometer MCR300 (Anton Paar, Graz, Austria) with cone plate geometry (CP 50-1) at 25 °C. The minimum gap size was 0.1 mm, and with a cone angle of 1°, the gap is much larger at the edges. Apparent viscosity measurements were performed after preshear treatment at shear rate 0.02 s^–1^ for 180 s and rest at 0 s^–1^ shear rate for 120 s. The shear rate was logarithmically increased from 0.1 to 1000 s^–1^ and the flow behavior was reported as apparent viscosity as a function of shear rate. Rotational oscillation measurements of the storage modulus, *G*′, and loss modulus, *G*″, were made. A frequency sweep from 100 to 0.01 rad s^–1^ at constant strain 1%, and a strain amplitude sweep from 0.1 to 1000% at 1 rad s^–1^ angular frequency, was measured to observe the range of linear viscoelastic response. All measurements were made on fresh samples just after preparation.

## 3 Results and Discussion

### 3.1 Zeta Potential and DLS

**Figure 1:**
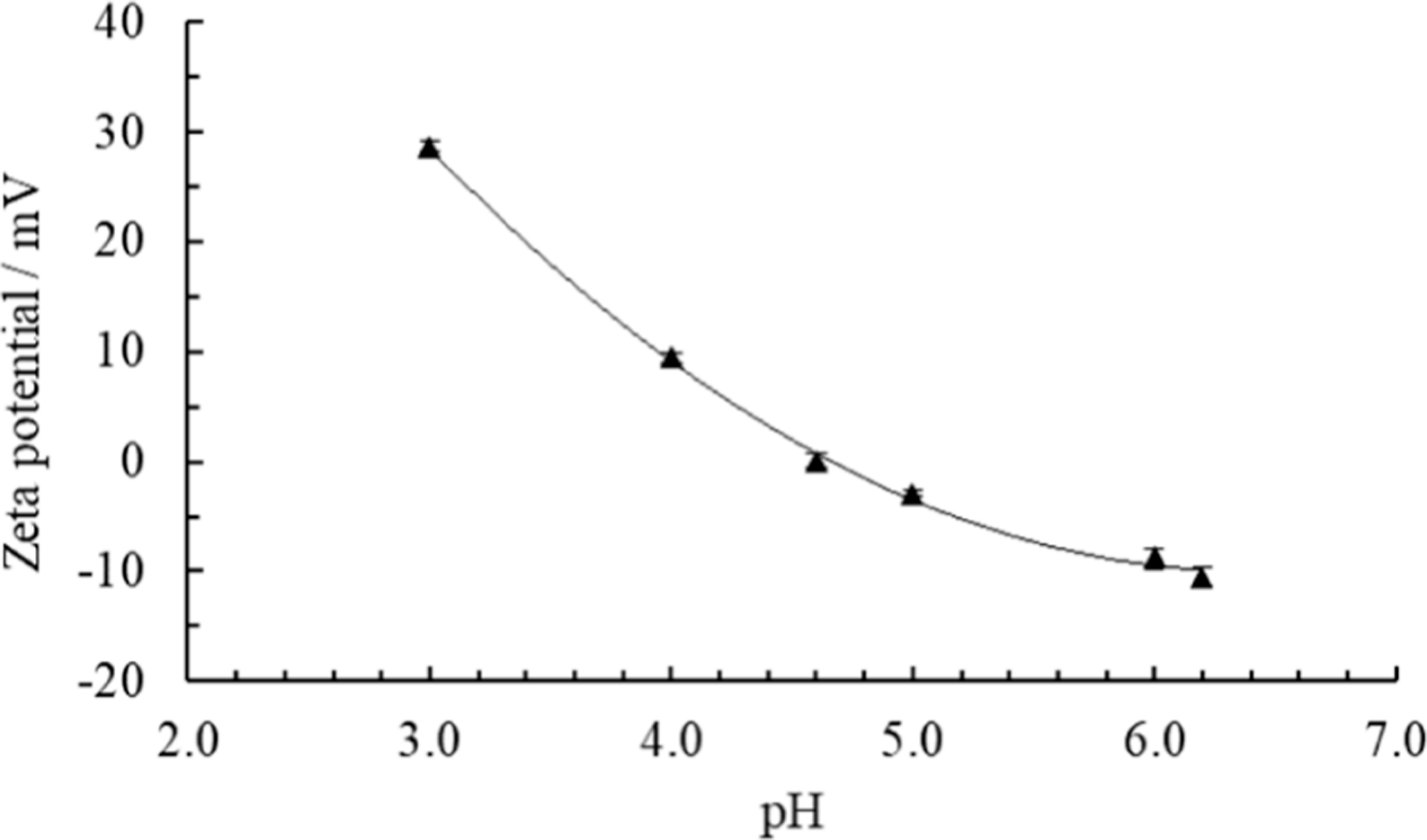
Zeta potential vs pH for pea protein dispersions in citrate buffer. The isoelectric point is at pH 4.67.

Pea protein dispersions (0.025% w/v) were prepared in 0.1 M citrate buffer at pH 3.0–6.2. The zeta potentials of the dispersions are shown in Figure 1. The highest value was determined at pH 3.0 where the amino groups were protonated and the net zeta potential decreases toward the isoelectric point. The high zeta potential at pH 3 suggests that the pea protein molecules are likely to repel each other and less likely to form aggregates. The positively charged amino groups in the pea protein dispersion and the negatively charged carboxyl groups equaled at pH 4.67 by a second-order polynomial fit, as seen in Figure 1. It has been reported that the isoelectric point for pea protein is at pH 4.6,^5^ pH 5.60,^21^ and pH 4.25.^22^ It is important to notice that the isoelectric point is not the same for all components in the pea protein material, and different fractions of globulins and albumins give different results. The isoelectric point for globulins is reported as pH 4.5 and for albumins as pH 6.^23^ This suggests that the pea protein is composed of mainly globulins, as also confirmed by earlier reports of 65–80% globulins.^5^ The isoelectric precipitation technique to extract pea protein is most commonly used at a pH around 4.5,^24^ which corresponds well with our results for the isoelectric point. The surface potential for pea protein dispersed in deionized water at neutral pH was determined as −13.0 ± 0.8 mV, which is a slightly lower net potential than −21.0 to −20.9 mV previously reported by Karaca et al. at neutral pH.^25^ They reported the zeta potential with 10 mM sodium phosphate buffer, which can explain the difference. Other reports include the zeta potential of 10% w/w oil emulsions as prepared with 0.5% w/v pea protein as −16.3 ± 0.1 mV.^26^

Protein solubility in aqueous solution is related to the zeta potential and the pH, and the lowest values are reported around the isoelectric point.^27−29^ The water-soluble fraction of pea protein isolate at neutral pH has been reported to range from 29.5 to 90.4% w/w, depending on the protein composition, extraction method, and treatment.^8,22,25,26,30^ The pea protein used in this study is likely on the lower end due to the commercial processing that enhances protein denaturation and aggregation during the isolation process.^31^ According to the Osborne classification, the globulin fractions are soluble in salt solution, as opposed to albumins which are soluble in water.^32^ A greater globulin fraction, as suggested by the zeta potential measurements, may also contribute to the lower water-soluble protein fraction.

**Figure 2:**
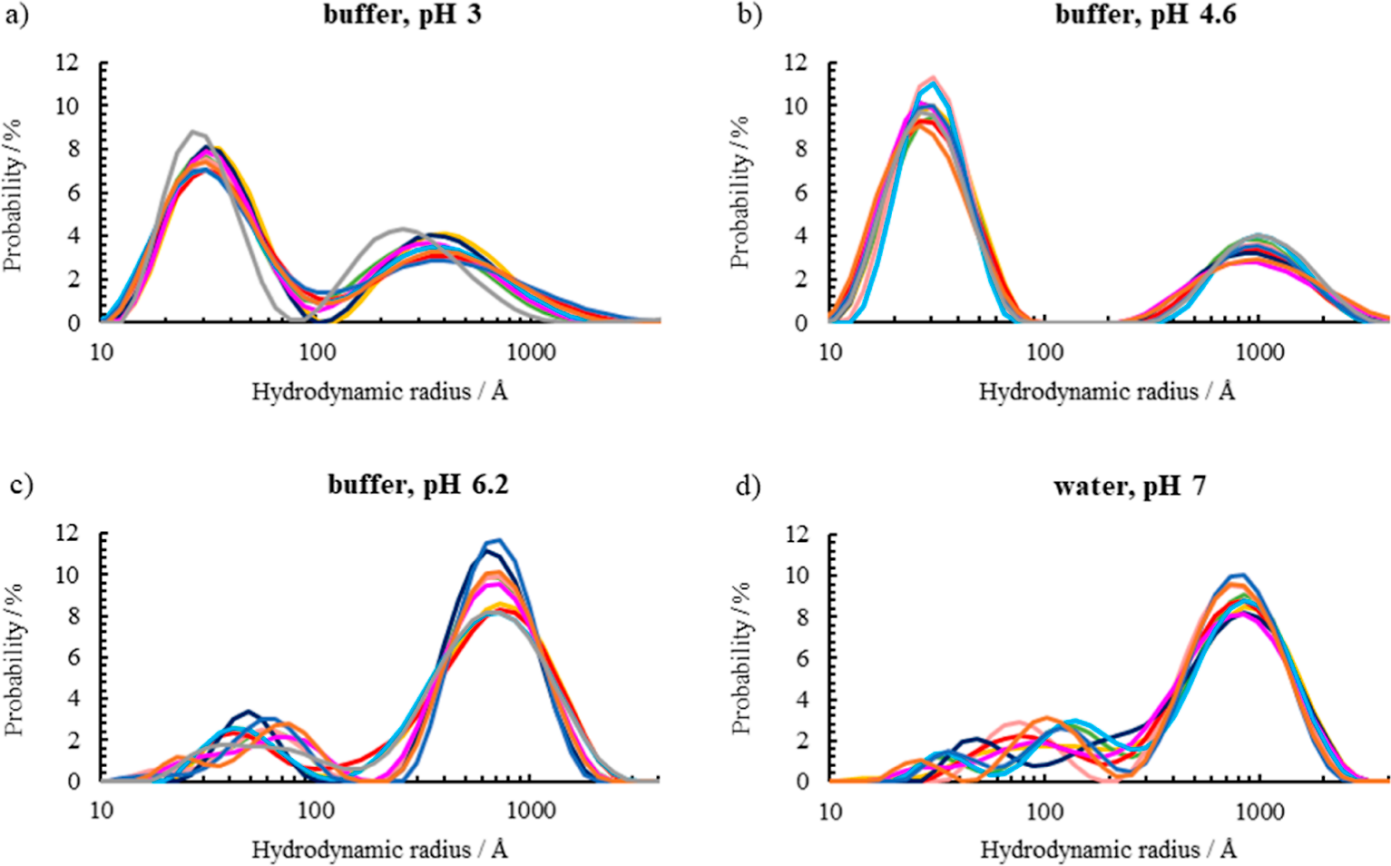
Particle size distributions weighted by scattered light intensity for pea protein dispersions at (a) pH 3, (b) pH 4.6, (c) pH 6.2 with buffer, and (d) neutral pH in water. The different lines show ten repeated measurements on the same sample. The samples show high polydispersity and multimodal populations. A general trend of increasing fraction of small particles at pH 3 and 4.6 is observed. The larger aggregates are smallest at pH 3 and biggest around the isoelectric point at pH 4.6.

The particle size distributions of pea protein in buffer and in water as measured by DLS at pH 3, 4.6, and 6.2 and at neutral pH are shown in Figure 2. The size is presented as radius distribution curves weighted by scattered intensity, and the volume weighted probability curves are shown in Figure S1 in the Supporting Information. The fractions of particles with smaller radii are higher at acidic pH 3 and around the isoelectric point, pH 4.6. The average hydrodynamic radius of the smaller particles at these pH values is 30 Å. The size and polydispersity of the smaller protein particles at pH 6.2 and at neutral pH are greater as seen by the shift to a larger radius of the probability curve and the variability among samples. The multimodal distributions with a smaller particle fraction in addition to bigger aggregates are present for all samples. The biggest aggregated pea protein material, 1000 Å, is present in the sample at pH 4.6 at the isoelectric point. Smaller aggregates, 400 Å, are found in the sample at pH 3 but the volume fraction of large protein particles is quite small at low pH. Aggregates of 700–900 Å are observed in the samples at pH 6.2 and at neutral pH with water. The polydispersity of particles and aggregates is important for the stabilization mechanism. The larger aggregates contribute mainly to the stabilizing layer covering the emulsion droplets as opposed to forming a dispersion in the aqueous solution due to their low Brownian motion. The dissolved smaller particles in the water phase contribute to steric hindrance and viscosity increase.

### 3.2 Physical Stability

**Figure 3:**
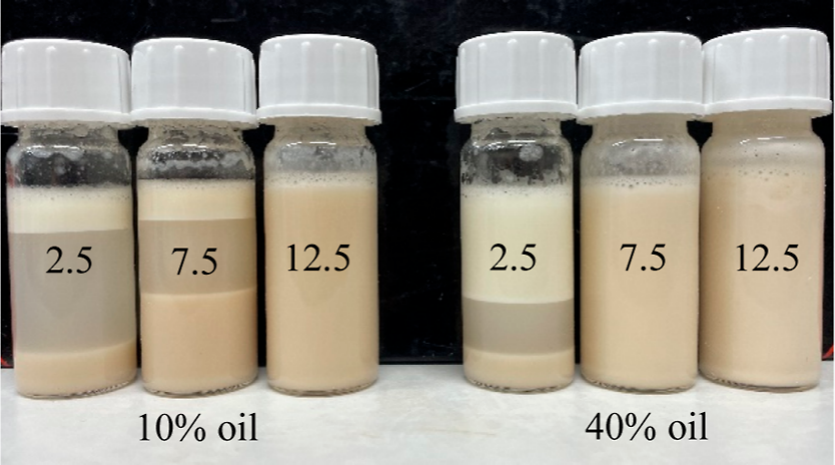
Photograph of the visual observation of stable and phase-separated emulsions. Example samples are shown from left to right: 10% v/v oil and 2.5, 7.5, and 12.5% w/v pea protein, followed by 40% v/v oil and 2.5, 7.5, and 12.5% w/v pea protein.

**Figure 4:**
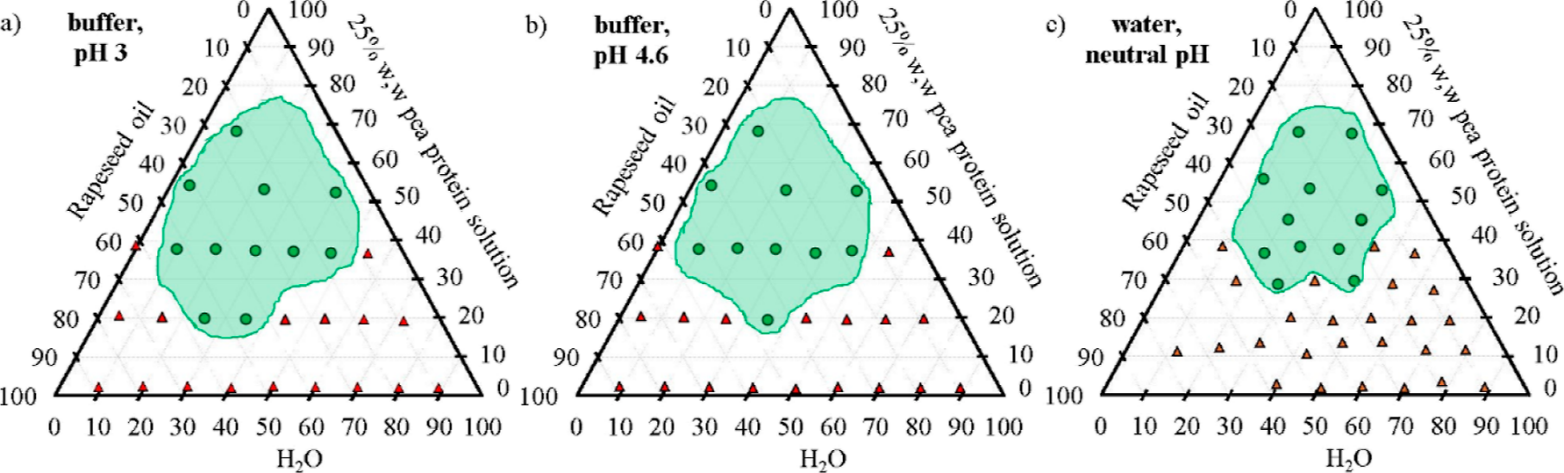
Stability ternary maps of emulsions at (a) pH 3 with sodium citrate buffer, (b) pH 4.6 with sodium citrate buffer, and (c) neutral pH with water. The corner points represent 100% w/w water, oil, and pea protein dispersion (25% w/w in water), respectively. The green circles are representative of visually stable emulsions after 7 days of storage at 22 °C, and the red triangles represent samples where phase separation occurred. The identified regions of stability are marked in green.

The visual appearance of stable and phase-separated emulsions after 7 days of storage is shown in the photograph in Figure 3. The visual appearance after 14 days of storage is shown in Figure S3. Emulsions at neutral pH after 7 days of storage show a single-phase region of stability for a high pea protein concentration of >5% w/w, at intermediate oil fractions of 30–50% w/w as shown in Figure 4c. For the lower oil fractions, stable emulsions are found with higher pea protein concentrations >12% w/w. The stable emulsions are characterized by a high viscosity, cream-like, homogeneous appearance and do not show any sign of creaming of oil droplets or sedimentation of pea protein due to the low molecular movement. The unstable emulsions were defined as samples that separated into two or more layers. One could argue that the phase-separated samples are metastable in the different parts of the phase diagram. The compositions of the two separated phases have not been studied further but are likely to be related to the reported stable compositions of high internal phase oil emulsions and low oil content, such as the studies of 80% v/v oil emulsions and 0.5% w/w pea protein in solution investigated by Li et al.^13^ and 5% w/w oil emulsions with 7.5–10% w/w protein in solution studied by Yerramilli et al.,^33^ respectively. The appearance after a few hours did not change significantly compared to storage after 7 days, as the emulsions reach a state of metastable equilibrium similar to earlier studies of the creaming stability.^30^ The destabilization rate was higher for low pea protein emulsions and with the increase of emulsifier concentration, the viscosity and stability increased. Investigating samples with a pea protein concentration >20% w/v was determined as outside the scope for this project as the excess protein has other functions than acting as an emulsion stabilizer. The high pea protein concentration of >5% w/v to produce stable emulsions is more than expected to cover the droplets. Even, assuming a droplet radius of 1 μm, calculations suggest that less than 1% w/v small pea protein particles are sufficient to form a monolayer to cover the interface for a 40% v/v oil emulsion. At concentrations ≥12% w/v, gelling effects with pea protein have been reported.^34^ The additional protein, which does not adsorb at the interface, could form a network that increases the viscosity and enhances the stability with gelling effects.

The pH effect on emulsion stability after 7 days of storage is seen in Figure 4. No significant differences in emulsion stability were identified at pH 3 and pH 4.6 (isoelectric point) but the samples at neutral pH showed a slightly smaller region of stability and faster phase separation. It has been suggested previously that protein-stabilized emulsions are the least stable around the isoelectric point because of large volume-mean droplet size, low percentage of adsorbed protein, and high creaming index.^35^ The present results with stability largely independent of pH suggest that the stabilization mechanisms are not strictly correlated to either the size of pea protein particles or charge effects.

As reported in our earlier review,^4^ the main effects for emulsion stability with pea protein are pea protein content and extraction process and the emulsion preparation technique is reported to have minor impact on storage stability. A systematic study of how pea protein-stabilized emulsions are affected by preparation methods such as high shear or high-pressure homogenization, sonication, and microfluidization has to our knowledge not been performed, and data points of stability from different studies are not always reported in a comparable way. By comparing two high shear preparation processes: that of a food blender and a homogenizer, no significant difference of the stability was observed as seen in Figure S2. The independence of storage stability on pH confirms that as long as some type of high-shear dispersing unit is used for emulsion preparation, the resulting droplet size, protein size, polydispersity, or charge does not seem to be the main parameters influencing stability.

### 3.3 X-ray Scattering Results

**Figure 5:**
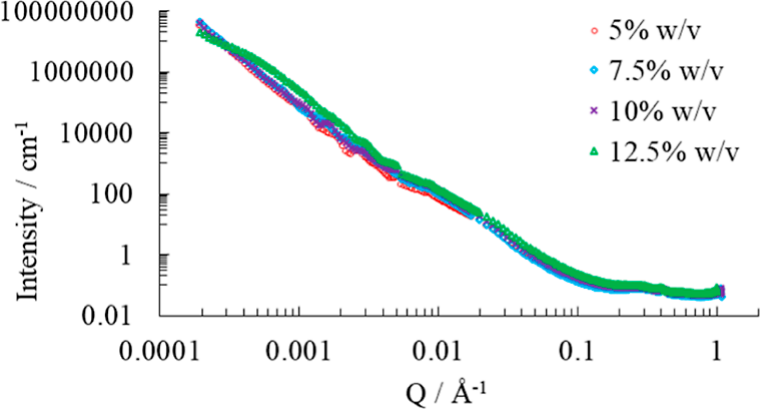
Merged desmeared USAXS and SAXS curves for emulsions with 40% v/v oil and 5, 7.5, 10, and 12.5% w/v pea protein.

Desmeared and merged scattering curves of visually stable emulsions with 5–12.5% w/v pea protein and 40% v/v oil, as measured by USAXS and SAXS, are presented in Figure 5. The broad *Q* range, 0.0002 to 1 Å^–1^, and intensity range covering 9 orders of magnitude correspond to real space structures of fractions of nanometers to several micrometers. The smooth curves without sharp maxima are an effect of polydisperse samples. The polydispersity is also seen in the light scattering results and confirmed with microscopy data, as seen in Figure S4. The momentum transfer region between 0.001 and 0.005 Å^–1^ contains many small oscillations. These should be treated carefully, as they arise due to noise and uncertainty in the measurements at the largest scattering angles of the USAXS measurements rather than being sample characteristics when presented as deconvoluted data. For this reason, it is preferable to further analyze data by fitting a model with an appropriate slit smearing to the measured data rather than trying to fit the deconvoluted data directly.

**Figure 6:**
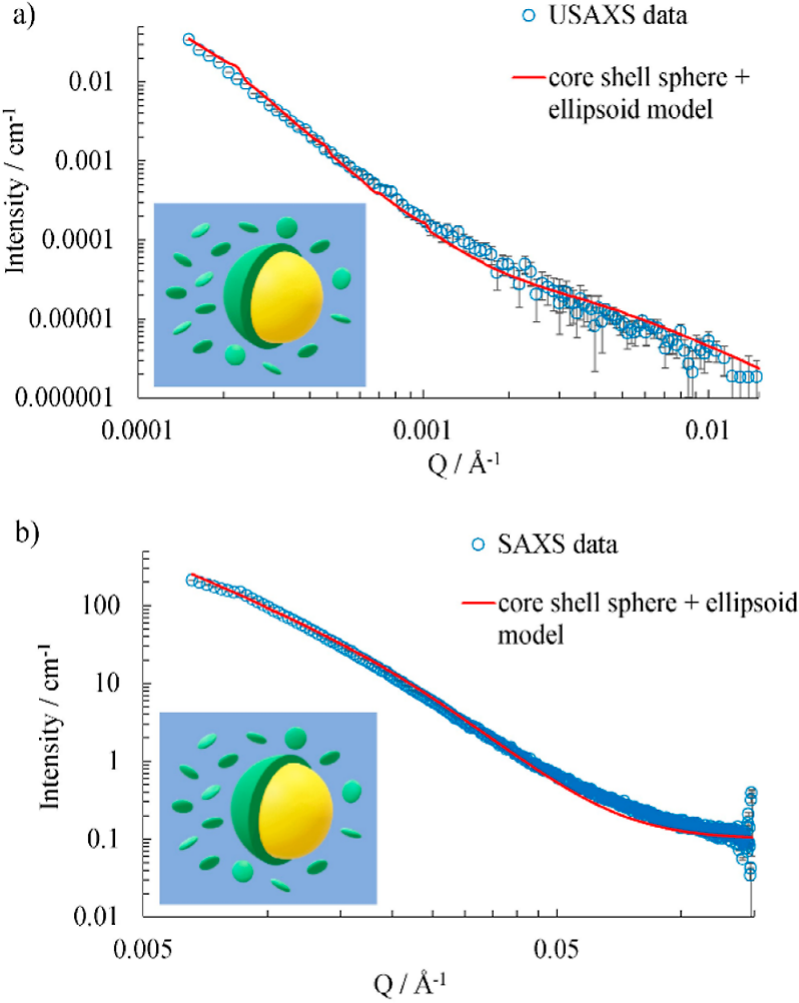
Model fits of (a) slit smeared USAXS data and (b) pinhole smeared SAXS data. A core–shell sphere model for the emulsion drop combined with ellipsoids that model the extra protein is plotted. The emulsion consists of 40% v/v oil stabilized by 7.5% w/v pea protein, and the relative volume fractions of the two models are 0.4 and 0.05, respectively. The insets show schematic representations of the model (not to scale).

A model fit to the USAXS and SAXS data for a sample with 40% v/v oil and 7.5% w/v pea protein over the *Q* range 0.0002–0.15 Å^–1^ are shown in Figure 6. The fit consists of a model that combines both core–shell spheres that represent adsorbed protein at the interface of oil droplets in water^36^ and ellipsoids for extra dispersed protein material in the aqueous phase.^37,38^ The scattering length density of the aqueous phase was fixed at 9.41 × 10^–6^ Å^–2^ and the relative volume fractions of the two models were fixed as 0.4 as the volume fraction of oil and 0.05 as the approximate amount of nonadsorbed pea material. The model parameters are shown in Table 2. The model suggests that about two-thirds of the pea protein material exists as ellipsoidal particles dispersed in the aqueous phase, which is in addition to the remainder present in a 700 nm-thick shell surrounding the oil droplets with radius 15.8 μm. This shell thickness corresponds to an overall concentration of about 2% w/v of protein. The polydispersity of the oil droplets was modeled as a Gaussian distribution. The dispersed pea protein ellipsoids with polar and equatorial radii 41 and 240 Å have a scattering length density of 11.1 × 10^–6^ Å^–2^ as hydrated protein in the aqueous phase. The scattering length density of the denser pea protein material in the shell surrounding the emulsion droplets is 12 × 10^–6^ Å^–2^. The results are in reasonable agreement with the size distributions obtained from DLS. The differences could be explained by some alteration of the size distribution for protein that has been homogenized during the preparation of the emulsion. The model suggests that the smaller particles of pea protein material are dispersed in the continuous aqueous phase, and the bigger aggregates are attached to the oil/water interface. The thickness of the interfacial layer has not been determined clearly in previous studies of pea protein systems. Although we cannot determine if the protein has been denatured, it is clear that there is a thick layer, much larger than the individual protein molecules, at the oil surface.

**Table 2:** Model Parameters for a Core–Shell Sphere and Ellipsoid Combined Model Fit

core–shell sphere + ellipsoid model parameters	
USAXS solid angle scale factor / sr	1.2 × 10^–6^ ± 5 × 10^–7^
SAXS background / cm^–1^ sr^–1^	0.1 ± 0.01
USAXS background / cm^–1^ sr^–1^	5.0 × 10^–7^
volume fraction of ellipsoids	0.05 ± 0.03
ellipsoid polar radius / Å	41 ± 5
ellipsoid equatorial radius / Å	240 ± 5
ellipsoid scattering length density / 10^–6^ Å^–2^	11.1 ± 0.5
solvent scattering length density / 10^–6^ Å^–2^	9.41 ± 0.01
distribution of ellipsoid polar radius (std. dev./mean radius)	1.0 ± 0.1
distribution of ellipsoid equatorial radius (std. dev./mean radius)	1.0 ± 0.1
volume fraction of droplets	0.40 ± 0.05
droplet radius / μm	15.8 ± 1
shell thickness / Å	7000 ± 100
droplet scattering length density / 10^–6^ Å^–2^	8.68 ± 0.02
shell scattering length density / 10^–6^ Å^–2^	12.0 ± 0.5
solvent scattering length density / 10^–6^ Å^–2^	9.41 ± 0.01
distribution of droplet radius (std. dev./mean radius)	1.0 ± 0.1
distribution of shell thickness (std. dev./mean thickness)	1.0 ± 0.1

The ambiguity of the model is related to whether the adsorbed layer is a uniform shell or consists of interacting particles. A more densely packed, uniform layer of protein or a higher scattering length density for protein particles gives similar effects. Our core–shell sphere model treats the protein as well-defined entities with a homogeneous scattering length density, but the possibility of a gradient scattering length density from the oil droplet core center to the continuous water phase could be considered. Scattering of particle-stabilized emulsions has been fitted with a “raspberry model” by Larson-Smith et al.^39^ The degree of hydration of the protein is difficult to assess with X-ray experiments, and details of the arrangement of the protein are not clear. However, variation of the D_2_O/H_2_O ratio in the aqueous phase and neutron experiments could in future studies give further information about the model. Significant information from the experiment fit is the presence of nonadsorbed pea protein material, as a simpler model of just core–shell droplets would not give a good fit. The details of the model parameters should be treated cautiously, as the ellipsoid model may be similar to a flat cylinder or an interconnected network of disc-like protein aggregates. The high polydispersity of the ellipsoidal radii makes a range of different shapes. The indication is that the importance of free protein in addition to the adsorbed material is crucial, but more work is needed to resolve the details of which protein components are contributing for each. A few studies have been reported involving separation of various different proteins such as globulin-, albumin-, legumin-, or vicilin-rich fractions.^40−43^ Kornet et al.^42^ reported that globulin-rich fractions are better as emulsion stabilizers, whereas albumin-rich pea protein is more effective as foam stabilizers. Kimura et al.^43^ investigated 7S and 11S globulins from various plants and found that the globulins from pea were poor emulsion stabilizers, whereas 7S globulin from fava beans gave good emulsion stability.

**Figure 7:**
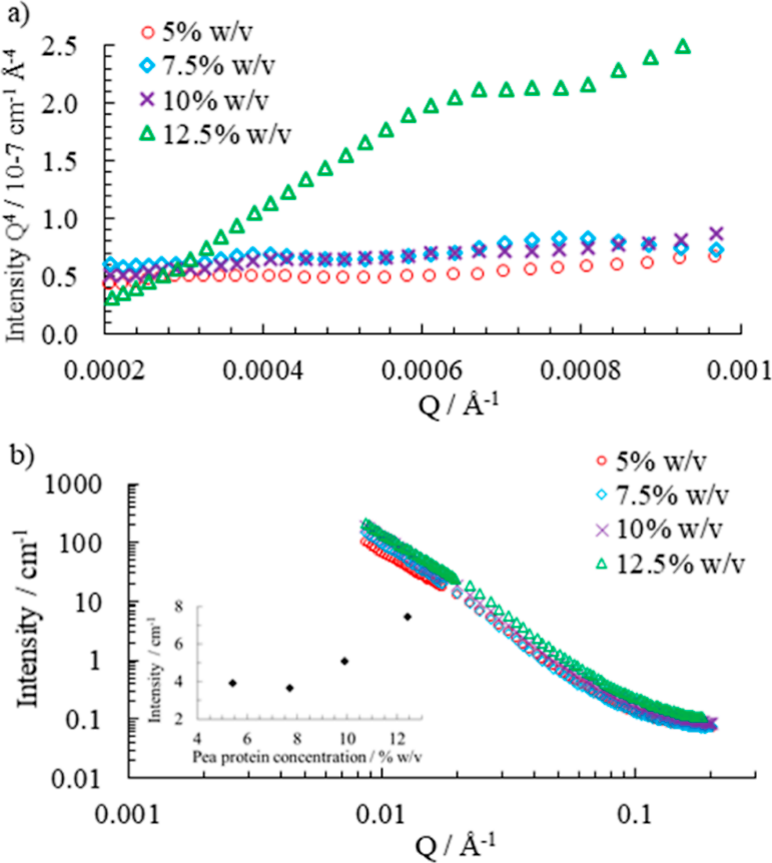
(a) Porod plot, *IQ*^4^ vs *Q*, at low *Q* values for emulsions with 40% v/v oil and 5, 7.5, 10, and 12.5% w/v pea protein. (b) SAXS data for the same samples over the *Q* range of 0.005–0.2 Å^–1^ and inset of intensity at 0.03 Å^–1^ as a function of pea protein concentration.

Due to the possible uncertainty or ambiguity in the model fit, some analysis of the scattering curves that is independent of any details of a structural model is useful. The data show a Porod region with approximately *I* ∼ *Q*^–4^ at low *Q* and a broad peak centered around 0.03 Å^–1^, as seen in Figure 7. The Porod law is an asymptote of *I* for values of *Q* that are large compared to the reciprocal of the size of the regions of heterogeneous composition. The specific surface area averaged within the sample volume, *S*, can be estimated directly from the scattering using the Porod law^44^ with
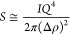
4where *I* is the scattered intensity, *Q* is the scattering vector, and Δρ is the difference in scattering length density between the two phases (i.e., oil and water). For an oil-in-water emulsion, this would represent the surface area of oil droplets divided by the total sample volume. The specific surface areas are determined for the samples with 5–10% w/v pea protein and they are presented in Table 3. The scattering length density was taken as 9.41 × 10^–6^ Å^–2^ for water and estimated as 8.68 × 10^–6^ Å^–2^ for the rapeseed oil based on the chemical composition^45^ and measured density. The specific surface area decreases with a higher protein concentration, which indicates the presence of smaller droplets. The radii of the spherical droplets are related to the specific surface area of the sample as
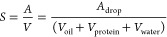
5where *A*_drop_ = 4π*r*^2^ is the area and *V* is the sum of the volumes of the different components per oil drop. It should be noted that the small particles would not contribute significantly to the Porod law scattering in the range of small momentum transfer that was used for the calculations. The discrepancy in the droplet radius from the model fit described in Table 2 arises from the evaluation of the Porod scattering. A correction factor depending on the polydispersity and the dimensionality is calculated and applied to the Porod law calculations.^46,47^ The resulting value of 15.3 ± 2 μm is close to the model fit of 15.8 ± 1 μm. The higher values compared to those obtained from the analysis of the optical micrograph shown in Figure S4 are likely effects of the ambiguity in identifying the droplets in the micrograph. Notably, the irregular shape of the smaller entities suggests that those are unlikely to be liquid oil droplets. Large aggregates of protein modeled in the SAXS analysis as the polydisperse ellipsoids may account for the low average radius. Furthermore, the analysis of the micrograph provides a number average rather than values calculated from surface scattering that provides markedly lower values for a broad distribution. The change in the scattering data for the sample with 12.5% w/v pea protein in Figure 7a suggests that there are smaller droplets, and the Porod region is at higher *Q* values in the region of highest USAXS measurements.

**Table 3:** Fitted Power Law Parameters in the Porod Region for *Q* Values below 0.001 Å^–1^^a^

pea protein / % w/v	oil / % v/v	power law slope	specific surface area / cm^–1^	radius / μm	radius corrected for polydispersity / μm
5	40	-3.8	15,400	0.78	20.3
7.5	40	-3.9	20,400	0.59	15.3
10	40	-3.8	19,400	0.62	16.1

a Values of the calculated specific surface area and corresponding radii are tabulated. The radii derived directly from the specific surface area and also corrected for the large polydispersity with the correction factor 26^46^ are shown. Data for samples with 40% v/v oil stabilized by 5–10% w/v pea protein.

The broad peak centered at around 0.03 Å^–1^ corresponds to real-space structures of size *d*, given roughly by
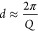
6and estimated as 210 Å. This correlation is evidently related to the protein, as the intensity varies with concentration. The approximately linear increase of intensity for the broad peak shown in Figure 7b suggests that this feature is an effect of the protein alone. These trends suggest two features of the emulsion structure. First, the droplet size is decreased at higher protein concentrations, which creates more surface area and hence more material giving rise to interface scattering. For other emulsion systems such as those with dairy protein, this behavior has been discussed. The globular whey protein, β-lactoglobulin, has been suggested as dissolved excess protein in the aqueous phase in addition to the formation of a single layer at the oil/water interface.^46^ Due to the low solubility of pea protein in water as discussed in Section 3.1 and the different pea protein components, the pea protein may be more likely to form a thick layer with multiple pea protein molecules around the droplets. Aggregation is likely to take place, as indicated by the light scattering results in Section 3.1. The repeatability of samples is good, and data are reproducible between samples prepared on different occasions as seen in Figure S5. Even for different preparation techniques, the structures are similar, as seen from the results in Figure S6.

### 3.4 Rheology

The viscoelastic properties of emulsions are dependent on several parameters: apart from the composition of the material, the strain rate and strain amplitude can significantly alter the observed behavior. As is common to many complex fluids, large strain amplitudes often show significant changes in properties that can arise from alterations in the structure of the sample. In order to see the effects clearly, we show example data for the emulsion with 40% v/v oil and 7.5% w/v protein in Figures S7 and S8. This composition was chosen as it is in the center of the region of stability. These initial results guided the choice of a strain amplitude of 1% for the frequency sweep measurements.

Oscillatory shear measurements were used to determine the storage modulus *G*′ and the loss modulus *G*″. A frequency sweep was made from 100 to 0.01 rad s^–1^ at a constant strain amplitude of 1%, and a strain amplitude sweep was made at 0.1–1000% at 1 rad s^–1^ angular frequency. Data for a sample with 40% v/v oil and 7.5% w/v protein are shown in Figures S8 and S9. The high *G*′ that represents an elastic response is dominant at low strain rates but the material behaves more like a viscous fluid at high strain rates as is seen from the relative values of *G*′ and *G*″. In many applications, the high shear rate regime is important. Further description is focused on the behavior in this region.

**Figure 8:**
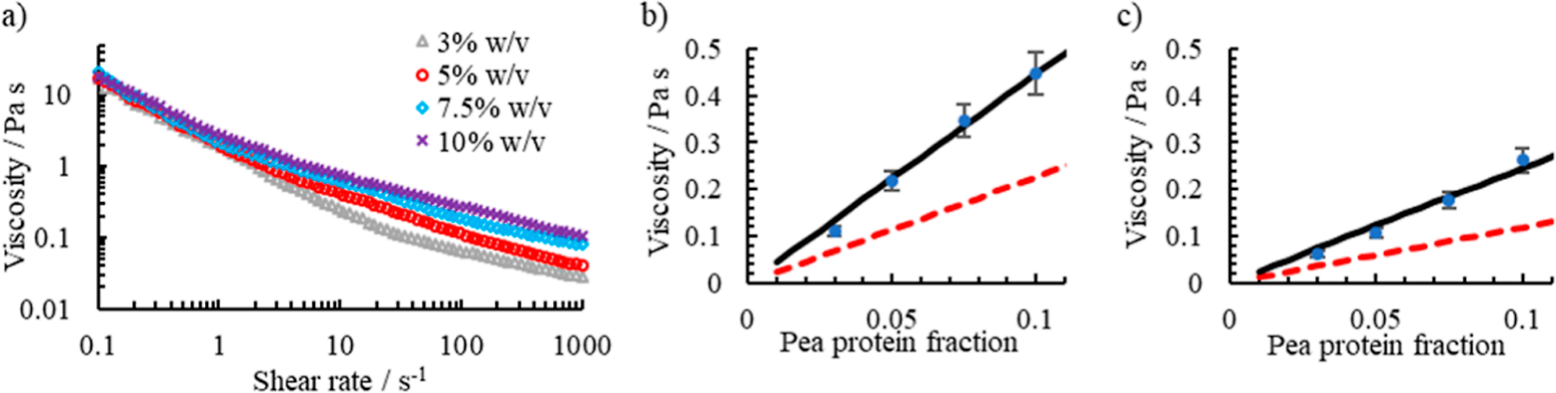
(a) Viscosity as a function of shear rate for emulsions with 60% v/v water and 40% v/v oil stabilized by 3, 5, 7.5, and 10% w/v pea protein. Viscosity at shear rate (b) 30.5 s^–1^ and (c) 110 s^–1^ as a function of pea protein concentration for dispersions with 60% v/v water and 40% v/v oil. The red dotted lines are derivatives from the Krieger–Dougherty model (eq 7) based on the dispersed oil phase. The linear fits to the viscosity as a function of pea protein concentration, black lines, have gradients double those obtained in the Krieger–Dougherty model.

**Figure 9:**
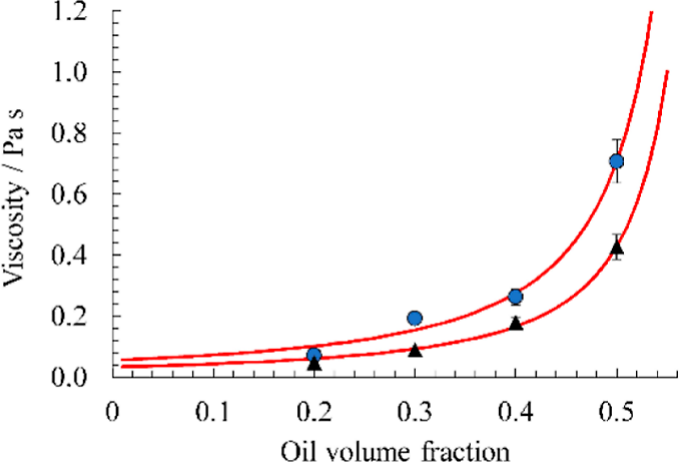
Viscosity at shear rate 110 s^–1^ as a function of oil volume fraction with 7.5% w/v pea protein (black triangles) and 10% w/v pea protein (blue circles). The red lines are fits of the Krieger–Dougherty model with the parameters discussed in the text.

The apparent viscosity of the pea protein emulsions was measured under continuous rotation at shear rates of 0.1–1000 s^–1^. The apparent viscosity decreased with increasing shear rate, as shown in Figure 8a. The viscosity as a function of the oil fraction was modeled by the Krieger–Dougherty model and is expressed as
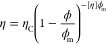
7where η is the viscosity of the emulsion system, η_C_ is the viscosity of the continuous phase, ϕ is the volume fraction of the dispersed phase, ϕ_m_ is the maximum phase volume, and [η] is the intrinsic viscosity coefficient taken as 2.5. The fitted parameters depend on the shear rate. Example fits of this model at the shear rate of 110 s^–1^ for pea protein concentrations of 7.5 and 10% w/v are shown in Figure 9. η_C_ was fitted as 0.033 and 0.055 Pa s, respectively, and ϕ_m_ was fitted as 0.62 for both samples. The maximum phase volume of the continuous phase is in the range of those found in other studies for particulate dispersions.^48^ The high viscosity is important for emulsion stability as the region of stability clearly shows stability at lower pea protein concentration only at the intermediate oil dispersed phase. The lower viscosity at high shear rates is probably an indication of distortion of the emulsion droplets and overall structure, and hence, the hysteresis in the behavior is observed.

The increase in viscosity with the pea protein concentration arises with a small change in the total volume fraction. It is helpful to compare this change to the gradient of the fitted Krieger–Dougherty curve for the sample at the specific oil fraction. Example data showing an approximately linear increase of viscosity with pea protein concentration are shown in Figure 8b. The derivatives for shear rates 30.5 and 110 s^–1^ at oil volume fraction 0.4 and pea protein concentration 7.5% w/v are 1.94 and 0.18 Pa, respectively. The viscosity increase with added protein is more than that which would arise from the addition of a similar volume of oil as the dispersed phase. The best linear fit to the increase in viscosity as a function of the pea protein volume fraction is double that of the gradient of the Krieger–Dougherty model fitted to the dispersed oil fraction. The factor of 2 indicates that the effective volume fraction of the protein is increased and the impact on viscosity with increasing pea protein is large. The approximate factor of 2 in the effect of the extra dispersed protein suggests that it occupies more space or is more hydrated than other materials. This observation also corresponds with the lower scattering length density found for this component in the fits to the SAXS data. Direct comparisons to previous studies are difficult as models of emulsions are usually modified to fit the specific data. The high pea protein concentration investigated in the current rheological study, which was determined by the range of stable compositions, has not been investigated before to our knowledge.

The changes in viscosity with volume fraction for emulsions have been reviewed in respect of theoretical models and experimental data by Pal^49^ and although there is broadly similar behavior for many systems, details of the maximum packing fraction can depend on the possible structures of the dispersed phase. In this respect, the distortion under shear of the oil droplets might be important, and there are models that account for the viscosity ratio of the fluids. However, these do not discuss the likely anisotropic shape of the droplets when subjected to continuous shear or the elastic and viscous properties of the stabilizing film at the surface. For the present system with a large amount of protein, these may be very significant. The polydispersity of the oil droplets would also significantly alter the maximum packing density of spheres.

The shear-thinning behavior for emulsions made with pea protein has been reported previously by Peng et al.,^30^ Zhang et al.,^50^ and Sridharan et al.^51^ Peng et al. investigated pea protein emulsions with 0.1–0.3% w/v pea protein and 10% v/v oil and reported a low apparent viscosity presumably due to the low volume fraction of oil and pea protein. Zhang et al. studied the viscosity of 1.0% w/v pea protein emulsions with 20% w/w oil at various different pH and salt concentrations and showed that samples were shear-thinning and the viscosity followed an Ostwald–de Waele relation given by
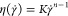
8where η is the apparent viscosity, γ̇ is the shear rate, *K* is the consistency coefficient, and *n* is the power law index. This model is used for shear-thinning samples without a Newtonian plateau region. Their samples^50^ show a lower overall viscosity and a low value of the consistency coefficient compared to our samples due to the lower volume fraction of dispersed material. The present measurements of viscosity for samples with a higher oil content show more marked shear-thinning behavior and a lower value of the power law index than what is presented in their study. Their suggestion is that a higher value of *K* and a lower value for *n* may be due to flocculation. Sridharan et al. explored 0.2–0.3% w/w pea protein emulsions in 10% w/w oil and found the apparent viscosity to be of the same order of magnitude as the study by Zhang et al. They also reported a similar behavior for pea flour and pea protein-stabilized samples with the same total protein content.

### 3.5 General Discussion

The multiple measurement techniques that have been used to probe protein in solution (DLS and electrophoresis) and emulsions (rheology and SAXS) provide a consistent picture of the emulsions. Overall, the scattering data give clear ideas about the emulsion structure and the location of the protein. The model-free analysis demonstrates the presence of both big emulsion droplets and smaller components of the protein material. The model fit that combines core–shell spheres to represent drops in the emulsion with protein at the interface and flat ellipsoids corresponding to a significant fraction of dispersed protein gives a fuller picture of the emulsion structure. Bigger aggregates are attached to the oil surface in a thick layer. Further proteins are located in the continuous phase. The observation in the maps of compositions that relatively large amounts of protein are needed to make stable emulsions leads to ideas as to the role of the excess above the minimum needed for a layer at the oil drop/water interface. The excess might be contributing to depletion effects and thus increase stability. Further possibilities would be that particular components of the isolate, distinguished either by the type of protein or by particle size, are the effective stabilizers in the bound layer. The difference in stability as seen at different pH values is interesting and indicates that the state of dispersion or the solubility of the protein causes significant effects.

## 4 Conclusions

The study has identified a wide range of compositions for stable oil-in-water emulsions with pea protein that is much more extensive than that found in a survey of the results that have been reported previously.^4^ It is apparent that for emulsions with relatively large amounts of oil from 30 to 50% v/v, about 5% w/v or more of pea protein isolate is required to prepare stable systems. This is in excess of what is needed to form a thin uniform layer of small particles at the interface. The stable emulsions can be prepared over a range of pH values from 3 to neutral. It is interesting and significant that the stability is not particularly correlated either with the overall isoelectric point of the protein, where it is likely to form insoluble particles, or to pH far from pH 4.6 with good protein solubility and possible charge stabilization. This contrasts with some other model emulsions, particularly those prepared with apolar oils such as alkanes where significant charge effects have been observed.^52^ In that study, the polarity of the oil is seen as important in reducing the effects of charge. The different states of the protein, the variation in droplet size, and the charge variation at different pH are shown not to be crucial parameters for stability. The large amount of protein required for stability is likely necessary as only a fraction is present as interfacial material at the various different pH values that have been investigated. The X-ray scattering shows that there is an interfacial protein layer of 7000 Å composed of dense pea protein material and that the excess protein is highly hydrated and dispersed in the aqueous phase. The fits of a proposed structure to the USAXS and SAXS data over a wide range and using absolute units for intensity allow for the entire composition of the sample to be included in the model that includes both the interfacial layer and extra dispersed protein. The droplets in the emulsion are very polydisperse, as seen in the optical micrograph and in the fit to the scattering data.

The rheology measurements with large increases in viscosity for relatively small increments in protein content, beyond those expected from volume fraction effects alone, tend to confirm the conclusions from scattering data that the excess dispersed protein is more hydrated than that found at the interface. The emulsions display shear thinning, as might be expected. The high viscosity can contribute to slower kinetic effects for coalescence of droplets and creaming, but the large amount and thick layer of protein at the interface is likely to be the dominant factor for stability as removing that from the drop surface is expected to have a high energy barrier.

By identifying the location and the role of different pea protein components as interfacial active materials or dispersed particles, it is possible to tailor properties such as viscosity and stability for different applications. This understanding will allow the future development of new food and nutraceutical products.

## Supporting Information Available

Complementary scattering, rheology, and microscopy data (PDF)
