# Supplementary material for: Understanding Stabilization of Oil-in-Water Emulsions with Pea Protein—Studies of Structure and Properties
Source: Langmuir. 2024 Jun 21;40(26):13386–96. doi: 10.1021/acs.langmuir.4c00540 (PMC11223488; doi:10.1021/acs.langmuir.4c00540)
Supplement: Supplementary file 1 — la4c00540_si_001.pdf [file la4c00540_si_001.pdf]

Understanding stabilization of oil in water emulsions with pea protein – studies of structure and properties - Supporting information

Eleonora Olsmats

Adrian R. Rennie

Macromolecular Chemistry, Department of Chemistry – Ångström, Uppsala University, Box 538, 75121 Uppsala, Sweden

Eleonora.Olsmats@kemi.uu.se

Adrian.Rennie@kemi.uu.se

Number of pages: 7

Number of figures: 9

Number of schemes: 0

Number of tables: 0

Table of contents

Dynamic light scattering 2

Physical stability.. 2

Microstructure 4

X-ray scattering.. 5

Rheology 6

Dynamic light scattering

The curves of probability by volume for the particle radius of pea protein in buffer solutions and in water as measured by DLS at pH 3, 4.6, 6.2 are shown in Figure S1. In this data representation, the contribution from objects with smaller radii are more pronounced than when weighted by scattered light intensity. This is seen in the disappearance of the peak for bigger aggregates seen in Figure 2. The trend of polydisperse samples is still seen. The mean average hydrodynamic radii weighted by volume at pH 3 and pH 4.6 are 22 and 20 Å, respectively. The values are bigger at neutral pH, with 33 and 50 Å in buffer at pH 6.2 and in water, respectively.

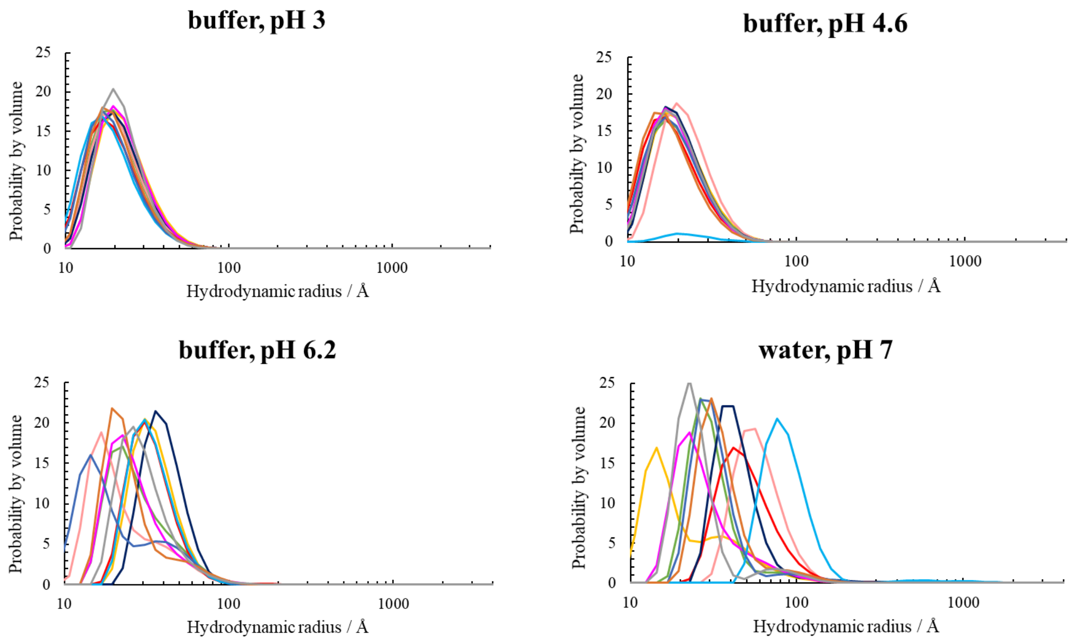

Figure S1. Particle size distributions for pea protein dispersions at pH 3, 4.6, 6.2 with buffer and pH neutral in water. The different multiple graphs are repeated measurements. The samples show high polydispersity.

Physical stability

In addition to the physical stability in response to pH, the influence of emulsion preparation technique was studied. Ternary stability diagrams for emulsions of various compositions prepared using a D1000 homogenizer with D1000-M5, 5 × 50 mm flat bottom probe (Benchmark Scientific Inc., Edison, NJ, USA) and a Blender 2 Go, Model BL3326B (Clas Ohlson AB, Insjön, Sweden) are shown in Figure S2. Similar regions of stability were identified for the samples prepared by the homogenizer and the food blender. This further confirms the idea that as long as some type of high-shear dispersing unit is used, specific values of the droplet size and polydispersity are not the main criteria affecting the emulsion stability. Other properties such as the pea protein composition and extraction process are more important for the emulsion stability. The greater variability for samples prepared with the food blender as seen in

the overlapping green and red markers in the stability map is a consequence of the less standardized preparation technique.

Stability of the emulsions was evaluated by their visual appearance after a fixed storage time. Figure S3 shows emulsions with 10 and 40% v/v oil after storage at 22°C for 14 days. The emulsions were made with water at neutral pH.

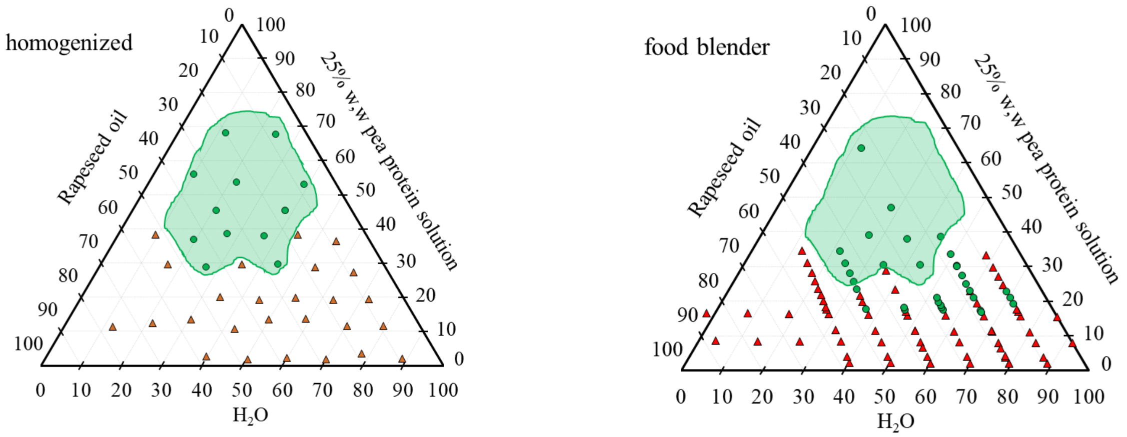

Figure S2. Ternary stability maps of pea protein emulsions after 7 days of storage for emulsions prepared by homogenization respectively by using a food blender. Green circles represent homogeneous emulsions and red triangles represent phase separated samples.

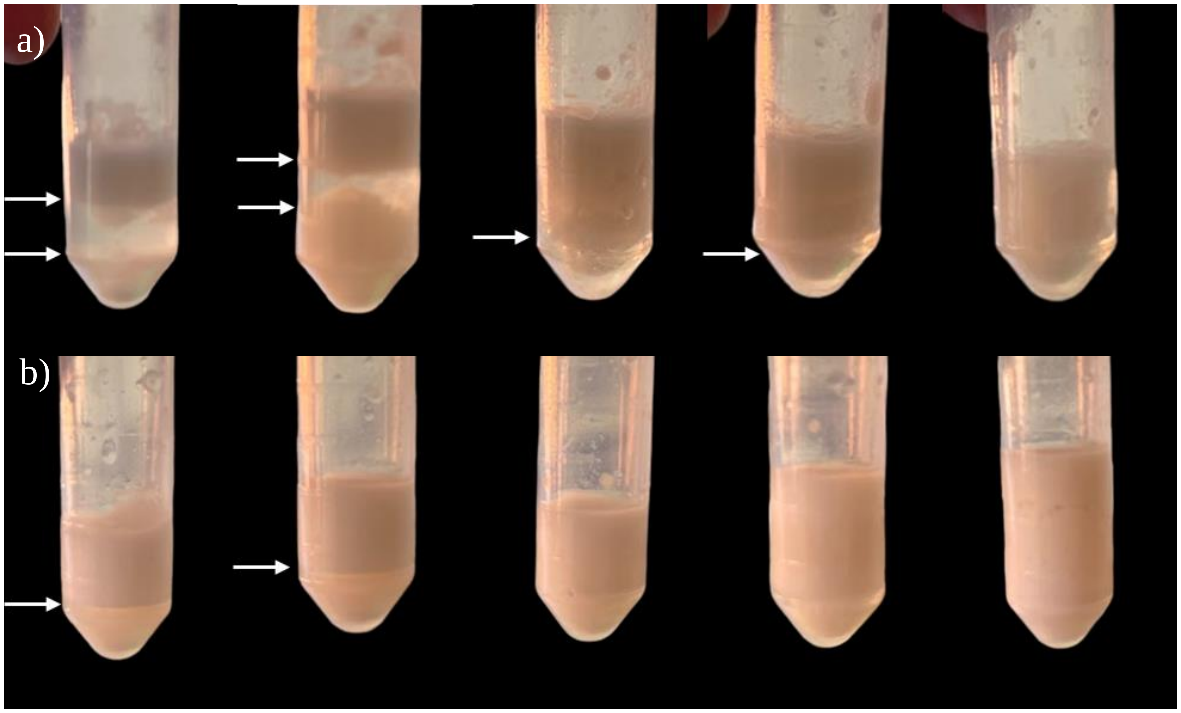

Figure S3. Photograph of the visual observation of stable and phase separated emulsions after 14 days of storage. Example samples are shown from left to right: 0.5, 3, 5, 7.5, 10 % w/v pea protein with a) 10% v/v oil and b) 40% v/v oil. White arrows are indicative of phase separation.

Microstructure

The micrographs in Figure S4 are obtained using a Leica DM4 B microscope in transmitted light with 100× magnification. The sample was placed between two glass slides to obtain an approximate thickness of 100 µm. Due to the droplet dimensions extending to tens of micrometers, the dimensions should be considered with care as the squeezing of droplets between the cover slip and microscope slide may alter the apparent diameter. Nevertheless, the images give a clear picture of the high polydispersity of the sample. A representative droplet size distribution for one micrograph is shown in Figure S4. The average droplet radius was determined as 2.5 µm. This number average is, as expected, considerably less than the values from scattering, both the model fit and the value calculated from surface scattering of 15.8 ± 1 and 15.3 ± 2 µm. This observation suggests a skewed number distribution of dominating smaller droplets or particles, with bigger droplets contributing heavily to the scattered intensity. The irregular shape of some smaller objects should be noted.

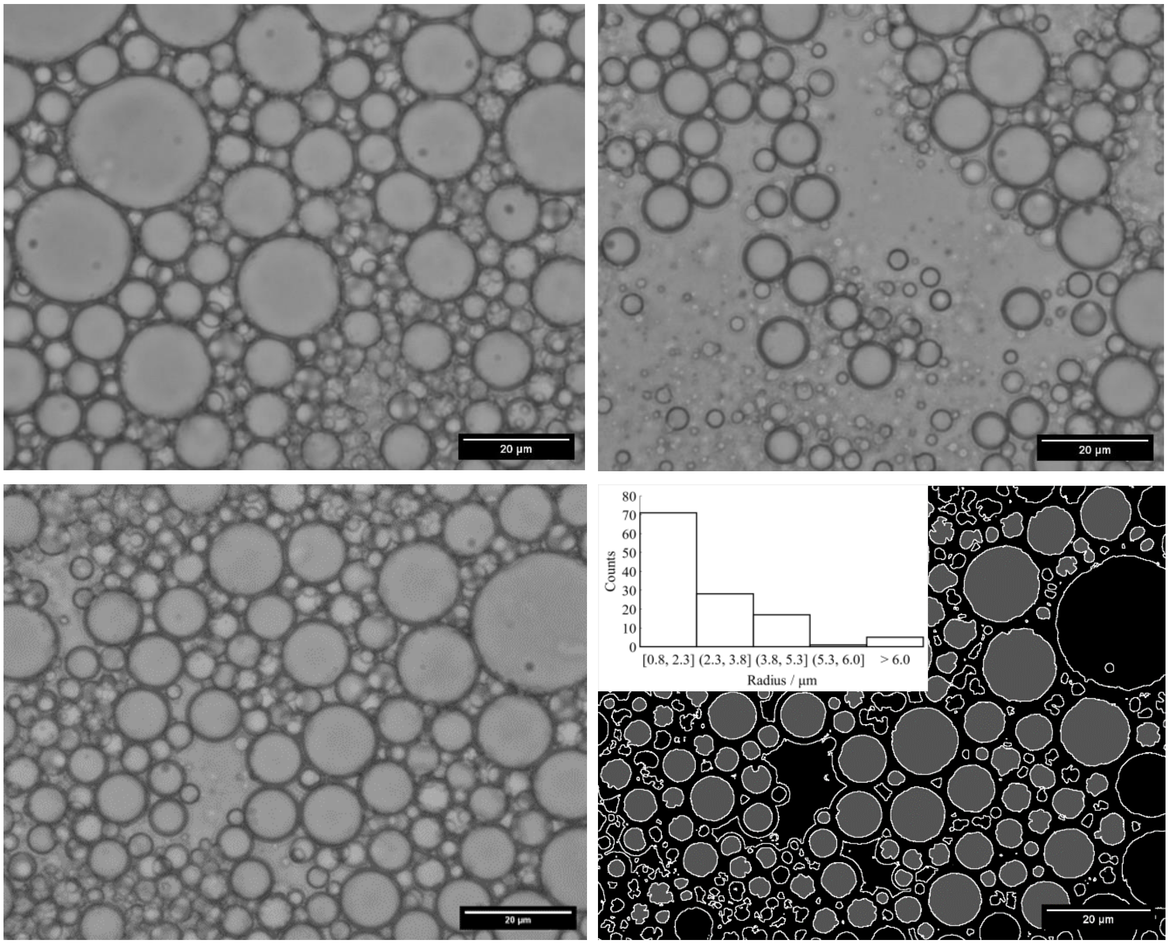

Figure S4. Optical micrographs of 7.5% w/v pea protein in 40% v/v oil-in-water emulsion for qualitative interpretation of the microstructure and droplet size. The emulsion is freshly produced. The scale bar is 20 µm. The bottom right image shows the droplet size distribution analysis using ImageJ software.

X-ray scattering

X-ray analysis and model fitting are useful techniques to understand the emulsion structure for a broad range of length scales. To obtain meaningful results, the repeatability of samples with reproducable structures is important. Five independent measurements at different dates and with different samples of the same composition have been performed and the overall characteristic features of the Porod region with approximatedly $I \sim Q^{-4}$ at low Q and a broad peak centered around 0.03 $Å^{-1}$ are similar. The data are plotted in Figure S4. There are some uncertainties in the thickness of the samples, as the Kapton windows used for sealing purposes are flexible in structure and hence the scattered intensity can vary. The sample thickness was estimated as 1.5 mm, but individual differences can make up uncertainties that we estimate of the order 1.2-1.8 mm thickness, corresponding to scale factor variation of about 25%. The small oscillations in the region of Q between 0.001 and 0.005 $Å^{-1}$ arise due to noise and uncertainty for the deconvoluted data in the largest scattering angles of the USAXS measurements. Therefore, these features are not reproducible between samples. Figure S5 shows scattering data for samples produced with a homogenizer and a food blender. The structures are similar and confirms the previous statement that the choice of high shear dispersing unit is not crucial for emulsion formation and structure.

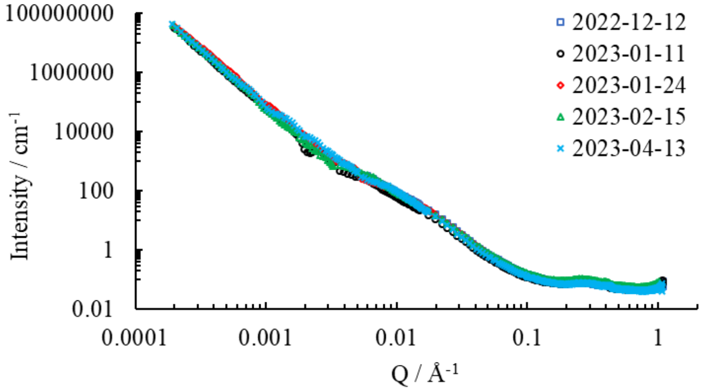

Figure S5. Measurements of USAXS and SAXS of 40% v/v oil different emulsion samples stabilized by 7.5% w/v pea protein. The results are reproducible and show the same broad peak feature and Porod region.

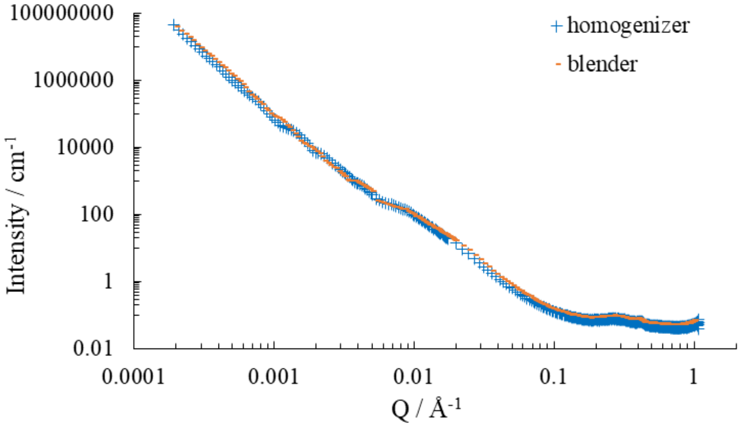

Figure S6. USAXS and SAXS plots of 40% v/v oil emulsions stabilized by 7.5% w/v pea protein prepared using a high shear homogenizer and a food blender. The results are reproducible and show the same broad peak feature and Porod region independent of preparation technique.

Rheology

Rotational rheological measurements of viscosity were made following the protocol of pre-shear at a shear rate 0.02 s^{-1} for 180 s and a rest period at 0 s^{-1} shear rate of 120 s, followed by logarithmic increase of shear rate from 0.1 to 1000 s^{-1}, and reversed. The difference in the viscosity curve for increasing and decreasing shear rate is seen in Figure S6. This result indicates that the emulsion structure and droplet formation undergo changes at high shear rates and that the structure is not recovered when returning to lower shear rates. Oscillatory rheological measurements were performed as amplitude sweeps at constant frequency of 1 rad s^{-1} and frequency sweeps at constant strain amplitude of 1%. Example curves for a sample with 60% v/v water, 40% v/v oil and 7.5% w/v pea protein are seen in Figures S7 and S8. The elastic response, G', is dominating in the low strain region, and the cross-over point, where the material starts to behave more like a fluid, is at 70% strain. The absolute values of G' and G" should be treated with caution for amplitudes larger than the linear viscoelastic regime of ~20% strain. These data are presented only to provide an indication of the linear viscoelastic regime and to indicate when structural changes start to occur in the emulsion.

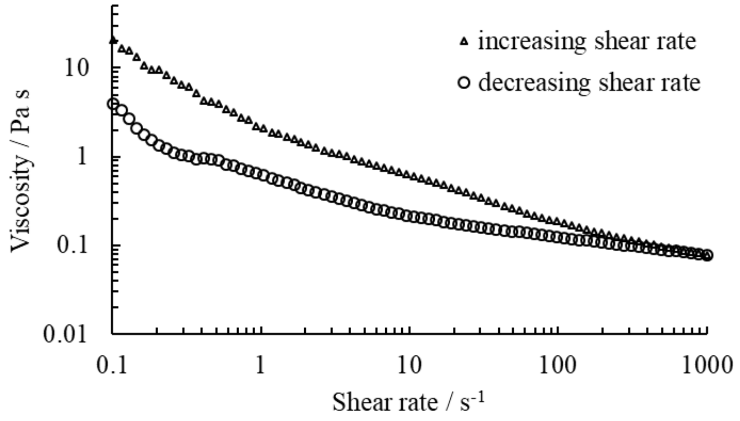

Figure S7. Viscosity as a function of shear rate for increasing shear rate followed by decreasing shear rate for an emulsion composed of 60% v/v water, 40% v/v oil and 7.5% w/v pea protein.

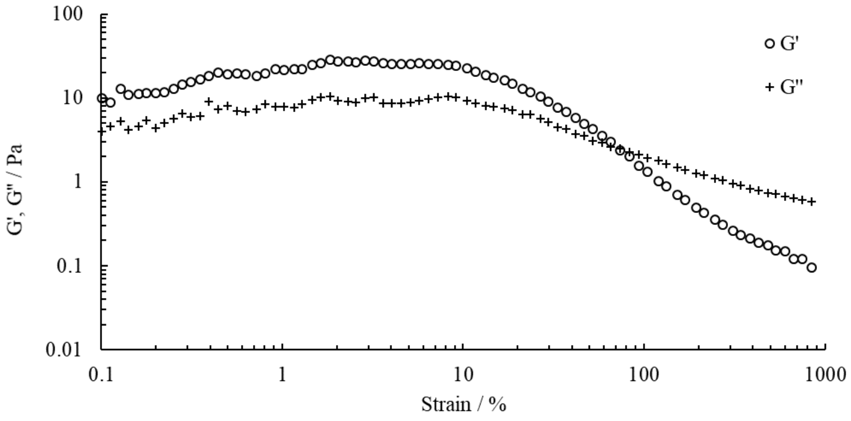

Figure S8. Amplitude sweep at frequency 1 rad s^{-1} for a sample with 60% v/v water, 40% v/v oil and 7.5% w/v pea protein.

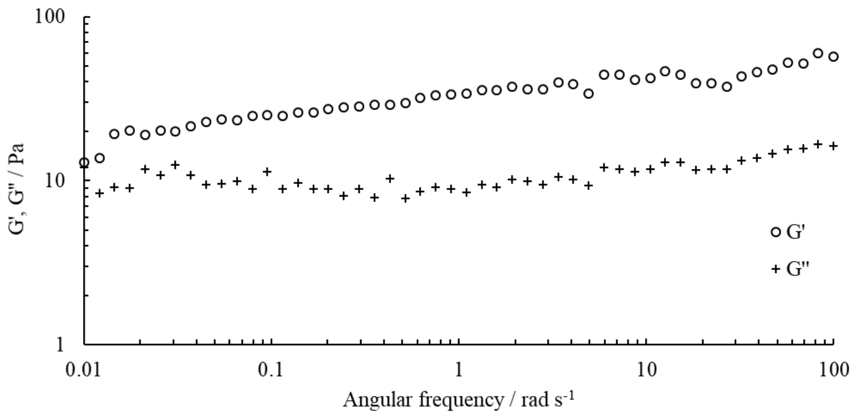

Figure S9. Frequency sweep at 1% strain for a sample with 60% v/v water, 40% v/v oil and 7.5% w/v pea protein.
